# Scaling up context-tailored clinical guidelines and training to improve childbirth care in urban, low-resource maternity units in Tanzania: A protocol for a stepped-wedged cluster randomized trial with embedded qualitative and economic analyses (The PartoMa Scale-Up Study)

**DOI:** 10.1080/16549716.2022.2034135

**Published:** 2022-04-12

**Authors:** Nanna Maaløe, Natasha Housseine, Jane Brandt Sørensen, Josephine Obel, Brenda Sequeira DMello, Monica Lauridsen Kujabi, Haika Osaki, Thomas Wiswa John, Rashid Saleh Khamis, Zainab Suleiman Said Muniro, Daniel Joseph Nkungu, Britt Pinkowski Tersbøl, Flemming Konradsen, Sangeeta Mookherji, Columba Mbekenga, Tarek Meguid, Jos van Roosmalen, Ib Christian Bygbjerg, Thomas van den Akker, Andreas Kryger Jensen, Morten Skovdal, Hussein L. Kidanto, Dan Wolf Meyrowitsch

**Affiliations:** a Global Health Section, Department of Public Health, University of Copenhagen, Copenhagen, Denmark; b Department of Obstetrics and Gynaecology, Hvidovre University Hospital, Hvidovre, Denmark; c Medical College East Africa, Aga Khan University, Dar Es Salaam, Tanzania; d Comprehensive Community Based Rehabilitation in Tanzania, Dar Es Salaam, Tanzania; e Temeke Regional Referral Hospital, Dar Es Salaam, Tanzania; f Mwananyamala Regional Referral Hospital, Dar Es Salaam, Tanzania; g Department of Global Health, George Washington University Milken Institute School of Public Health, Washington, DC, USA; h School of Nursing and Midwifery East Africa, Aga Khan University, Dar Es Salaam, Tanzania; i The PartoMa Project, Zanzibar, Tanzania; j Athena Institute, VU University, Amsterdam, Netherlands; k Department of Obstetrics and Gynaecology, Leiden University Medical Centre, Leiden, Netherlands; l Section for Biostatistics, Department of Public Health, University of Copenhagen, Copenhagen, Denmark; m Section for Health Services Research, Department of Public Health, University of Copenhagen, Copenhagen, Denmark

**Keywords:** Obstetrics, Africa, urbanization, intervention, co-creation, cost-effectiveness, stillbirth, perinatal death, respectful maternity care, low dose high frequency training, programme theory, de-colonizing

## Abstract

While facility births are increasing in many low-resource settings, quality of care often does not follow suit; maternal and perinatal mortality and morbidity remain unacceptably high. Therefore, realistic, context-tailored clinical support is crucially needed to assist birth attendants in resource-constrained realities to provide best possible evidence-based and respectful care. Our pilot study in Zanzibar suggested that co-created clinical practice guidelines (CPGs) and low-dose, high-frequency training (PartoMa intervention) were associated with improved childbirth care and survival. We now aim to modify, implement, and evaluate this multi-faceted intervention in five high-volume, urban maternity units in Dar es Salaam, Tanzania (approximately 60,000 births annually). This PartoMa Scale-up Study will include four main steps: I. Mixed-methods situational analysis exploring factors affecting care; II. Co-created contextual modifications to the pilot CPGs and training, based on step I; III. Implementation and evaluation of the modified intervention; IV. Development of a framework for co-creation of context-specific CPGs and training, of relevance in comparable fields. The implementation and evaluation design is a theory-based, stepped-wedged cluster-randomised trial with embedded qualitative and economic assessments. Women in active labour and their offspring will be followed until discharge to assess provided and experienced care, intra-hospital perinatal deaths, Apgar scores, and caesarean sections that could potentially be avoided. Birth attendants’ perceptions, intervention use and possible associated learning will be analysed. Moreover, as further detailed in the accompanying article, a qualitative in-depth investigation will explore behavioural, biomedical, and structural elements that might interact with non-linear and multiplying effects to shape health providers’ clinical practices. Finally, the incremental cost-effectiveness of co-creating and implementing the PartoMa intervention is calculated. Such real-world scale-up of context-tailored CPGs and training within an existing health system may enable a comprehensive understanding of how impact is achieved or not, and how it may be translated between contexts and sustained.

Trial registration number: NCT04685668

## Background

Globally, more than 300,000 women die each year during pregnancy and childbirth [1]. Moreover, 2.0 million stillbirths occur, half of which happen during birth, and 2.4 million newborns die during the first 28 days of life [2,3]. Many more survive with birth-related disabilities and trauma [4–6]. The vast majority occurs in the world’s poorest countries, and tragically, the numbers are forecasted to increase substantially as an indirect consequence of the COVID-19 pandemic [7].

Advocating for skilled birth attendance has been a central global strategy for decades to end this preventable burden of lost lives. While facility births are increasing, quality of care, however, often does not follow suit [8]. Due to inadequate human resources, infrastructure and equipment, a dangerous coexistence is apparent of maternity care that is ‘too little, too late’ (TLTL) and ‘too much, too soon’ (TMTS) [9]. This is particularly evident in urban, resource-constrained health systems where indicators of higher maternity care coverage compared to rural areas may be misleading; an urban disadvantage is reported from several low- and lower-middle-income countries (LLMICs) with higher maternal and neonatal mortality, urban poverty, lack of basic infrastructure and alarmingly congested health facilities [10–12]. For instance, the current rise in non-medically indicated caesarean sections (CSs) is an alarming TMTS concern, for which a major underlying cause appears to be TLTL surveillance and care during birth [9,13,14]. Hence, generating timely, evidence-based and respectful maternity care in low-resource settings is key to reaching Sustainable Development Goals (SDGs) for birth-related survival, gender equality, and improving working conditions and retention of the health workforce. Moreover, these transformations are crucial for societal development and poverty reduction (SDGs 1.1, 1.2, 3.1, 3.2, 3c, 5.1, 8.8) [15–19].

With this purpose, multiple large-scale, international clinical practice guidelines (CPGs) have emerged for low-resource settings, including electronic critical care pathways and other algorithms [9,20]. As the World Health Organization states, CPGs ‘are the fundamental means through which the Organization fulfils its technical leadership in health’ [21]. Such CPG development processes, however, typically rely exclusively on international experts within the medical field while neglecting representatives of those who are most familiar with the context and will have to use and live with the CPGs. Furthermore, little attention is paid to facilitate CPG development or adaptation at national and sub-national levels or to ensure effective implementation and rigorous evaluation. Consequently, frequent incompatibilities with local realities in under-resourced health systems limit actual CPG use. Moreover, it often results in demoralized health workers, drained resources and, paradoxically, unintentional harm to clinical practice [22].

### PartoMa pilot intervention

To bridge the CPG development and implementation gaps, the PartoMa pilot study in Zanzibar, Tanzania (2014–2018) showed how a co-created, multifaceted intervention of context-tailored CPGs with low-dose, high-frequency training was associated with promising improvements in care quality and perinatal survival (Box 1) [23–25]. CPG co-creation with Zanzibari birth attendants revealed that considerable changes had to be made to internationally established CPGs with regard to frequency of assessments, information load, and ambiguity. This is crucial to make CPGs feasible, safe, and easy-to-use for mostly young, inexperienced, and overburdened birth attendants with limited access to in-service training and supervision. The final PartoMa CPGs were reviewed and endorsed by an international panel of specialists [26]. After six years, the Zanzibar PartoMa intervention of CPGs and training continues to be used among Zanzibari health providers.

**Figure:**
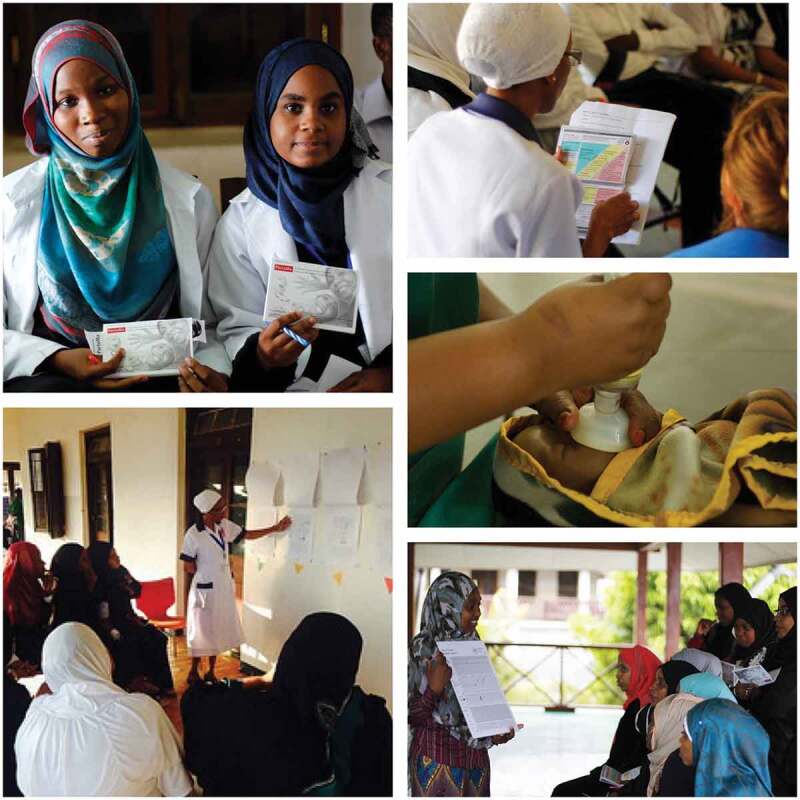

While the pilot study’s results are promising, the PartoMa intervention needs more robust implementation research: *i)* the intervention (CPGs and training) was contextually tailored for and evaluated in one maternity unit only and transferability needs assessment; *ii)* deeper understanding of the context and ‘active ingredients’ in the intervention and implementation strategy is needed; and *iii)* evaluation of cost-effectiveness is crucial for comparison with alternative investments in maternal health [27,28].

### Next step: PartoMa scale-up study

In response to the key targets of Tanzania’s health sector strategic plan [29], we set off to explore the scale-up of the PartoMa intervention in five urban maternity units in Dar es Salaam. This includes analysing associated processes, effects, costs, barriers, and facilitators. Furthermore, the study provides a broad situational analysis of the urban health system’s challenges in keeping up with an urbanization rate above 5% [11]. Notably, with more than half of the world’s births occurring in urban areas by 2030, such research into the access and quality of maternity care in urban maternal health is warranted [30].

Informed by the Standards for Reporting Implementation Studies (StaRI) [27] and the CONSORT Statement for reporting randomized trials [31], we here present the overall design for this mixed-methods co-creation and implementation study. The methodology for the qualitative components is briefly described, but is further elaborated in an additional study design paper published separately [32].

The overall study objective is:

To assess barriers, facilitators, effects, and cost-effectiveness of co-creating and implementing context-specific CPGs and low-dose, high-frequency training to improve quality of care and survival during childbirth in five urban, low-resource, high-volume maternity units in Tanzania.

The specific study objectives include:

(I)To carry out a mixed-methods assessment of care provision and experience of care in the five maternity units, focusing on present and past structures within which care is given, processes of care provision and birth outcomes. [Step I]
(II)To explore and develop necessary context-modifications for PartoMa CPGs and training to reflect birth attendants’ and labouring women’s needs and circumstances in the five maternity units, and to assess whether and how resources and experiences that stakeholders contribute are informing and steering the process. [Step II]
(III)To assess the effects of the context-modified PartoMa intervention on perceptions, knowledge and skills among birth attendants, quality of care provision, experience of care among women giving birth, and birth outcomes in the five maternity units, its cost-effectiveness, as well as opportunities and barriers in the process. [Step III]
(IV)To develop a framework for co-creating and implementing CPGs and associated training that may be relevant within and beyond maternal health. [Step IV]

## METHODS

### Study organization

The study is based on a collaboration between Aga Khan University, Tanzania, the non-governmental organization ‘Comprehensive Community Based Rehabilitation in Tanzania’ (CCBRT), University of Copenhagen, Denmark and VU University of Amsterdam, the Netherlands. The study team includes five PhD students and two postdocs with support from senior researchers and trained research assistants. The researchers’ backgrounds cover clinical obstetrics and midwifery, social sciences, implementation science, epidemiology, statistics, and health economics.

Since 2010, CCBRT has supported maternal and perinatal health care in 22 of Dar es Salaam’s health facilities, including the study sites, through training, infrastructure upgrades, and data strengthening initiatives [33]. Thereby, CCBRT will play a major role in data collection and facilitation of close collaboration with the hospitals’ administrations, the district medical officer and the regional administrative office for health.

The advisory board, which includes representatives from the Tanzanian Ministry of Health and international experts, will oversee study methodologies and alignment with national guidelines and policies. The advisory board will also assist in bridge-building for policymakers and other stakeholders.

### Study setting

Dar es Salaam is the largest and fastest growing city in East Africa. Its population is approaching six million and will become a megacity with more than 10 million inhabitants by 2030 [34]. In Tanzania, care coverage during pregnancy and childbirth is higher in urban areas than in rural areas, such as pregnant women reaching four antenatal care visits (63.8% versus 45.0%), proportion of births in health facilities (86% versus 54%), and population-based CS rates (12% versus 4%) [35]. This however, stands in contrast to a disadvantage in urban birth outcomes; in 2016, the perinatal mortality rate was estimated at 47 per 1,000 births in urban versus 37 per 1,000 in rural areas, and the 2012 consensus found maternal mortality ratios of 432 versus 336 per 100,000 live births [35,36].

As seen in other urban settlements of LLMICs, higher wealth inequality, urban poverty, lack of basic infrastructure, and congested health facilities are associated with such disadvantages [10,11]. While 47% and 49% of rural women and men own houses, either alone or jointly, this is the situation for 23% and 27% in urban areas. Fewer urban women complete primary school than in rural areas (47% versus 52%) and women’s employment rate is lower (66% versus 76%) [35]. Simultaneously, access to quality health care is compromised as the health system has not been able to follow suit with population increase [33]. Within maternity care, the extremely high patient flows combined with significant resource and staff shortages are likely to be key drivers of the dangerous combination of TLTL and TMTS care, as well as of disrespect and abuse during childbirth. As in other LLMICs, studies from urban maternity units in Tanzania report startling examples of how women’s basic human rights are violated. This has obvious implications for maternal and perinatal safety, including abandonment, non-dignified and non-consented care, and physical and psychological abuse [37–40].

Adding further to the complexity of providing maternity care in congested, resource-constrained facilities, urban birth attendants increasingly must manage an alarming double burden of co-morbidities, including infectious and cardiovascular-metabolic diseases. In 2016, 62% of the urban women still perceived malaria and HIV/AIDS as the most serious health problems in Tanzania. Simultaneously, urban women increasingly suffer from obesity, hypertension, and diabetes; 42% of the urban women in Tanzania are overweight (body mass index ≥25) versus 21% of their rural counterparts [35,41]. On top of this, the majority of COVID-19 cases occur in urban settings [35,42].

The PartoMa Scale-up Study will be conducted in five of Dar es Salaam’s government-owned maternity units, which provide comprehensive obstetric and neonatal care: Mwananyamala, Amana and Temeke are regional referral hospitals and Sinza and Mbagala Rangi Tatu are district hospitals (Figure 2). In 2019, they had been the five highest volume maternity units for more than a decade (annual average of 13,116 births per facility), and they jointly cared for approximately 40% of all urban and sub-urban births in Dar es Salaam [33]. These five sites enable a robust stepped-wedge design (please see power calculation below). At the same time, it was estimated to be realistic, considering the study’s available resources and the desire for more in-depth analysis.

Notably, the COVID-19 pandemic may have influenced the distribution of births in Dar es Salaam, which has likely led to an overall decrease of facility-births [7,43]. No Tanzanian data currently exist about possible COVID-19-related indirect consequences. Yet, modelling studies and emerging data from other LLMICs indicate how focusing resources to fight COVID-19 can further debilitate frail health systems and counteract improvements for maternity care, including raised maternal and perinatal mortalities [7,44]. In addition, introducing user fees for childbirth in the three regional referral hospitals in October 2019 might have influenced the distribution of births in the city. User fees entail that all women must pay 1–3 USD on admission as well as 11–32 USD for vaginal birth and 22–87 USD for CS. Women pay the lowest prices when they are referred from other facilities. The difference to the district hospitals is, however, partly balanced as women are expected to bring supplies for their birth or give a contribution (17–30 USD), which may not be required in the regional referral hospitals. There are, furthermore, additional fees in all five facilities for laboratory investigations, and for medications not available in the hospital. Notably, 90% of the Tanzanian women (age 15–49 years) in urban areas do not have any health insurance coverage [45]. Possibilities exist, however, for exemptions when women cannot afford fees or purchases.

Irrespective of potential declines in facility births, preliminary data indicate that the five study sites remain typical examples of overburdened, urban maternity units in LLMICs; they primarily serve women of lower socioeconomic status who live below the international poverty line, and each birth attendant is typically expected to attend to at least three to six labouring women simultaneously [11,30].

### Programme theory

The PartoMa intervention is a complex, multi-faceted clinical intervention, which is influenced by context in all stages, from co-creation to implementation and evaluation [28,46]. In particular, complexity arises from multiple interacting components (Box 1) and because birth attendants are co-creators as well as implementers and users of the intervention. Also, complexity stems from the intervention’s self-directed approach, which inevitably causes selection bias (seminar attendance, CPG use and supervision of others rely on individual motivation). The aim of the intervention is likewise complex as it seeks both to improve quality of care and birth outcomes directly, and more broadly to improve awareness of and skills in the development of contextualized CPGs and associated training for use within and beyond maternal health.

**Figure 1.**
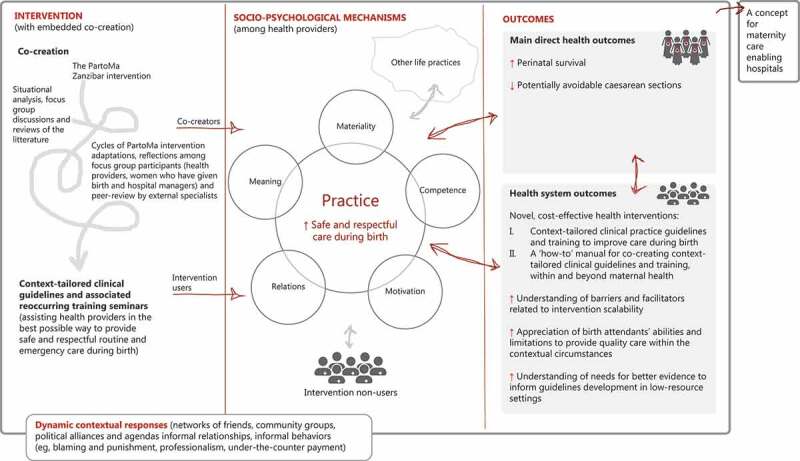
The PartoMa intervention’s programme theory. It is hypothesized that the intervention, with embedded co-creation, improves clinical practice and the desired health and health system outcomes through a reconfiguration of interacting mediators, which are divided into practice theory’s five analytical domains [47]: 1. *Meaning* (changed norms and values that circulate within the maternities, including an increased participatory/self-directed approach to development and use of guidelines and training, critical dialogue, teamwork and supervision); 2. *Materiality* (provision of PartoMa pocket booklets with guidelines, as well as changed use of existing medical equipment, medicines, infrastructures and the body); 3. *Competence* (increased understanding of clinical deficiencies and abilities, evidence- and context-informed re-negotiation of what is best possible practice, and increased clinical knowledge and skills in intrapartum care); 4. *Motivation* (Increased intrinsic motivation among health providers that enjoin and direct to use the intervention); 5. *Relations* (Increased sense of being heard and understood by intervention developers and colleagues, facilitation of a blame-free, social space for learning through critical dialogue and supervision). In addition, *other life practices* refer to social practices, such as family obligations, that may be influenced by the work environment. These hypothesized mechanisms are further unfolded separately [32].

To explore this complexity, a programme theory has been developed, which serves as a hypothesis of non-linear pathways through which the intervention interacts with interlinked behavioural, biomedical, and structural elements, leading to the desired aims (Figure 1) [27]. As a starting point, we applied experiences from the PartoMa pilot study in Zanzibar [23], and as described in detail separately [32], the programme theory was further modified through the application of ‘practice theory’ and workshops with the study team and representatives from the study sites [47]. Based on the programme theory, multiple quantitative and qualitative study methodologies have been selected. The model may undergo additional adaptations as findings from the situational analysis emerge.

**Figure 2.**
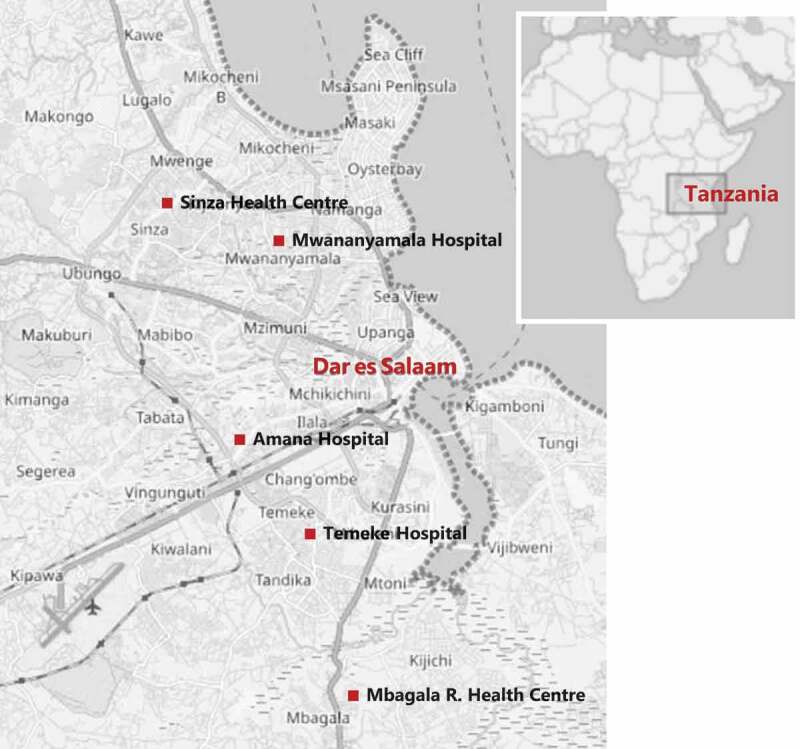
Map of the five maternity units in Dar Es Salaam, Tanzania. In 2019, they had been the five highest volume maternity units for more than a decade. *Source: open street map.*

#### I. Situational analysis

A positive outcome of the PartoMa Scale-up Study relies on interaction with and understanding of the contextual circumstances in which the PartoMa intervention is modified, implemented, and evaluated [28]. The main goals of the mixed-methods situational analysis are to quantitatively and qualitatively assess historical and current processes and quality of intrapartum care, including quality and use of routine clinical data, and to explore the broader context influencing this. This will inform context-modifications of the PartoMa intervention, provide rich descriptions of the context for external validity, and enable understanding of how best to generate accurate data for the evaluation.

##### A. Historical assessment

Through CCBRT reports and routinely collected data, the aim is to explore the influences of past conditions, such as capacity strengthening initiatives, changes in staffing or birth volumes and user fees [28]. This has resulted in a decade-long historical review of quality improvement, changes in workload and birth outcomes in the five study sites and neighbouring facilities of Dar es Salaam (2010–2019) [33]. An analysis of the years 2020–2021 will follow, including a focus on the COVID-19 pandemic and how it has influenced maternity care.

##### B. Quantitative assessment

The structure within which care is currently provided, the processes of care provision and birth outcome indicators will be assessed (Table 1) [48]. Structure indicators will be collected through observations, questionnaires for birth attendants and checklists for each facility. To assess the current processes of intrapartum clinical care and associated birth outcomes, a case-control study will be conducted of all intra-hospital perinatal deaths in 2020 (birthweight ≥2000 g), compared to randomly selected women with healthy perinatal outcomes (birth weight ≥2000 g, Apgar score ≥8, alive on discharge).

**Table 1.** Analytical framework for quantitative data collection during situational analysis and intervention evaluation. Process and outcome data will be collected through structured observations, criterion-based audits of case files, questionnaires and knowledge-skills tests. For all women and health providers, background characteristics will be recorded as well (women: age, parity, previous perinatal death, antenatal care attendance, date and time of admission/birth/discharge; health providers: age, educational level and years of experience with maternity care). The analytical framework for the qualitative research components is elaborated separately [32]

**Structure**	**Process of clinical care during birth**			**Direct health outcomes**
**Workload***(patient volume; numbers, composition and organization of providers; provider-to-labouring women ratios)* **Management /leadership structure** **Infrastructure and supplies***(physical organization of maternity units, essential medication, monitoring devices, birth equipment, disinfection facilities, personal protective equipment)* **Other ongoing interventions***(e.g. training, supervision, clinical practice guidelines, construction)* **Data quality***(registration of clinical assessments, maintenance and use)* **Referral systems**	**Clinical surveillance** Admission assessment *(history and physical assessment)* Partograph use Foetal wellbeing *(by foetal heart auscultation)* Labour progression *(by recordings of cervical dilatation and membranes)* Maternal vital signs *(blood pressure, pulse rate, temperature)*	**Intrapartum treatment** Caesarean section *(divided into medically indicated and potentially avoidable)* Prolonged labour *(incl. timely and safe use of artificial rupture membranes and oxytocin)* Severe hypertensive disorders *(incl. timely use of antihypertensives and magnesium sulphate)*	**Caring support** Communication *(incl. verbal and physical abuse)* Birth companionship and emotional support Respect and preservation of dignity *(e.g. privacy)* Pain relief Mobilization and birth position User fee structure	**Primary outcomes** Perinatal survival *(intra-hospital stillbirths, pre-discharge neonatal deaths within 7 days)* Potentially avoidable caesarean sections *(by assessment of care before caesarean section)* **Secondary outcomes** Perinatal outcomes *(perinatal deaths divided into 1000–1999 g and ≥2000 g, neonatal first 24 hours pre-discharge mortality, Apgar score <7 and <9, admissions to neonatal intensive care)* Maternal outcomes *(death, postpartum haemorrhage, perineal tear, uterine rupture, postpartum hysterectomy)* Mode of birth *(spontaneous vaginal birth, vacuum extraction, caesarean section)*
				**Health system outcomes**
	**Health providers’ perceptions**			**Cost-effectiveness** (incremental costs and effects related to the intervention, related to maternal complications, intra-hospital perinatal deaths, Apgar score <7 and potentially avoidable caesarean sections)
	Access to the intervention during clinical work Participation rates during PartoMa seminars Perceptions of the intervention’s relevance, effects and limitations			
	**Health providers’ competencies**			
	Knowledge in management of intrapartum care Partograph skills			

##### C. Qualitative assessment

A mix of methods will be employed (participant observations, individual interviews, and focus group discussions (FGDs)) with a range of actors (birth attendants, hospital leadership, and women who have recently given birth). The aim is to investigate how participants experience, interpret, and engage with current maternity care, with a particular focus on respectful care and intrapartum decision-making in the case of prolonged labour and mode of birth. Further methodological details are presented separately [32].

##### D. Mixed-methods Analysis of Data Quality and Use

The quality and use of routine clinical data will be analysed through translational steps from clinical assessments to case file recordings, from case files to hospital registers, from registers to policy level and from policy level back to the frontline. This analysis will include quantitative and qualitative study findings mentioned above as well as further direct, structured observations and reviews of case files and registers.

#### II. Intervention co-creation

##### Co-creation process

The PartoMa pilot intervention from Zanzibar, including CPGs and training, will be modified and possibly elaborated, in accordance with pregnant women’s and birth attendants’ needs and circumstances in Dar es Salaam [49]. Co-creating and ensuring timely updating of CPGs may be highly resource-intensive for each single maternity unit in low-resource settings and it causes confusion if health providers are often shifted between facilities [22]. Therefore, and in line with demand by the regional medical officer in Dar es Salaam, the aim is to co-create only one version for all five sites.

We plan for a dynamic co-creation process lasting approximately six months, which might undergo adaptations on the way to reach the best possible, locally acceptable, simple, and realistic CPGs and training materials [49]. Sampling of co-creators will be opportunistic, with primary co-creator groups being nurse-midwives and medical doctors from the study sites. In addition, the intervention will be reviewed by hospital, district and regional health managers, by representatives from health colleges in Dar es Salaam and by an external panel of international experts in midwifery, obstetrics, and neonatology. The research team, including focal persons from the study sites, will facilitate and participate actively in the process. In particular, they will conduct and present situational analysis, summarize evidence, and provide scientific literature where needed (e.g. oxytocin augmentation in low-resource settings), synthesize inputs from co-creators and update the guidelines and training content accordingly.

A first draft of the modified CPGs and training content will be developed based on the situational analysis and initial FGDs with birth attendants (around five groups with 8–12 participants in each group, divided into professional backgrounds). Through a new round of FGDs and individual assessments of guidelines and training content, health providers will share their perspectives, which will inform further modifications. The iterative cycles of feedback from FGDs, intervention modifications, and reviews will be conducted until co-creators and reviewers are satisfied with the intervention and implementation plan (i.e. there are no more shared concerns regarding specific CPGs, training components, or graphic presentation, which might be dangerously (mis-)used in clinical work or lead to mistrust or neglect of the intervention) [26].

Notably, we aim for co-creators and birth attendants to acquire ownership through repeated discussions where openness and perceived control of the process are central. We hope that the actors will invest themselves in the process, as ownership may be strengthened by self-investment. Moreover, as reflected in the program theory (Figure 1), we hypothesize that co-creation leads to ownership, which will be a central driver of successful implementation and sustainability [49].

##### Analyses of co-creation

Three levels of analyses will be done of the co-creation process: *a*. core components of the intervention that ‘survive’ between settings and modifications needed will be identified and compared to both the Zanzibari PartoMa pilot intervention and international CPGs; *b*. whether and how co-creators influence the co-creation process and experience this will be ethnographically assessed through participatory observations and in-depth interviews; *c*. costs of the co-creation process will be recorded to inform the cost-effectiveness analysis of the PartoMa intervention.

#### III. Intervention implementation and evaluation

**Figure 3.**
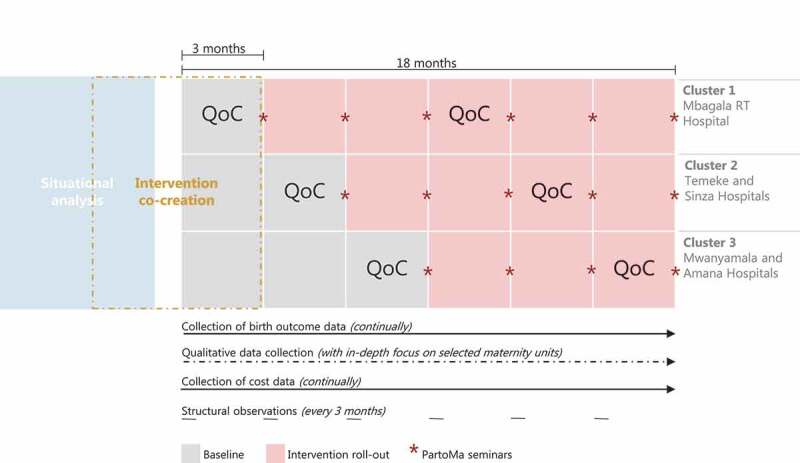
Overall implementation design for the pragmatic stepped-wedged cluster-randomized trial in five maternity units, divided into three clusters. Stars indicate when quarterly PartoMa seminars will be conducted in each facility. At the seminars, attendees’ perceptions and learning curves will be assessed. Quality of care (QoC) assessments through criterion-based audits and structured postpartum interviews with women will be conducted during baseline and the 7^th^ to 9^th^ intervention month at each maternity unit. Structured observations of infrastructure, equipment, data management and usage of the PartoMa guidelines will be conducted every three month. Birth outcome data, cost data and qualitative data will be collected continuously.

The overall implementation design will be a pragmatic stepped-wedged cluster-randomised trial with the five maternity units divided into three clusters receiving the intervention at cluster-level [50]. In a random order generated by R (R Core Team, 2017), maternity units will receive the intervention in three-month intervals. After enrolling in the third cluster, the intervention and evaluation will continue for additional nine months in all five sites (Figure 3).

##### Evaluation of direct health outcomes

Our primary direct health outcomes are perinatal survival (stillbirths with positive foetal heart upon admission and pre-discharge neonatal mortality during the first seven days among inborn babies weighing ≥1000 g) and potentially avoidable CSs (by criterion-based case file review of care preceding surgery). Our secondary direct health outcomes include additional perinatal indicators (perinatal deaths divided into 1000–1999 g and ≥2000 g, first 24 hours pre-discharge neonatal mortality, Apgar score <7, admission to neonatal intensive care unit), maternal indicators (death, postpartum haemorrhage, uterine rupture, postpartum hysterectomy), and mode of birth (spontaneous vaginal birth, vacuum extraction, CS). In addition, background characteristics will be recorded for all women (age, parity, previous perinatal death, date and time of admission and birth, and birthweight). Sociodemographic indicators such as marital, economic, and educational status are not available in birth registries.

Birth outcomes and mode of birth will be prospectively measured from baseline and until nine months after the PartoMa intervention is implemented in the last facility. Thus, data collection will be ongoing for 18 months (Figure 3). Data will be gathered twice a week on each of the five study sites, primarily from the intrapartum birth register. Key indicators will be cross-checked with the antenatal, surgical, neonatal and postnatal wards registers and with the death certificate counter book. For each study site, data will be double entered every fifth week to ensure that the accuracy of data retrieval remains above 95%. Women referred to the study sites prior to birth from non-study health facilities will be included, but their clinical care prior to admission will not be possible to assess. When referrals occur from one to another study site, data will be recorded on where the woman gave birth. Babies referred postpartum for intensive care will be recorded at the facility where the woman gave birth (Table 1).

##### Evaluation of quality of care (QoC)

Quantitative assessments of potential changes in the QoC during birth are divided into provision and experience of care [51], with sample size calculations based on findings from the situational analysis.

For care provision, a criterion-based audit of case files will be conducted at each facility during three baseline months and the 7^th^-9^th^ implementation months (Figure 3, Table 1). This analysis is limited to intrapartum management preceding CSs, including the rate of potentially avoidable CSs, and intrapartum management of severe hypertensive disorders. These focus areas suit our preliminary assessments of data availability and accuracy in case files. Moreover, they represent a major challenge in reaching beyond the co-existence of TLTL and TMTS care to save lives [9]. Within the pre- and post-intervals at each study site, all case files will be assessed during the weekly data collection to retrieve case files of: i. women giving birth by CS; and ii. women suffering from severe hypertensive disorders (systolic BP ≥160 and/or diastolic BP ≥ 110 and/or eclampsia) (Figure 3, Table 1). Both the analysis of potentially avoidable CSs and the analysis of severe hypertensive disorders will be based on audit criteria, which were previously successfully applied in Tanzania [25,52,53]. Notably, indicators and methods for auditing case files might be further modified based on the final results of the situational analysis.

To assess potential changes in women’s experiences of care, women will be invited to respond anonymously to a validated, structured questionnaire through phone calls 2 to 4 weeks after birth. Compared to interviews before discharge where women have yet to reflect on their experiences, this timeframe has previously proven relevant in the Dar es Salaam context to ensure adequate reporting [54]. In each facility, the assessment will be conducted during the three baseline months and the 7^th^-9^th^ implementation months. The questionnaire is developed based on a qualitative participatory approach consisting of iterative rounds of feedback from local and international stakeholders [37,38,55]. The main focus areas are overall satisfaction with care and occurrences of disrespect and abuse [32].

In addition to this quantitative QoC evaluation, a broader qualitative investigation will be conducted as described below. Furthermore, to understand potential changes in the underlying structures within which care is provided and received, including usage of the PartoMa CPGs, all five facilities will be visited every third month throughout implementation and evaluation to retrieve data using a structured questionnaire (Table 1).

##### Evaluation of birth attendants’ perceptions and learning

At each quarterly seminar, health providers will be asked to fill in two anonymous questionnaires:a Likert-scale evaluation of their experiences and perceptions of the intervention (after attendance of seminars) and a knowledge test (before and after attendance at seminars) (Figure 3). Participants’ perceptions, return rates, and learning over time will be followed using unique participant numbers. As no allowances are paid, attendance and return rates are considered indicators of interest and motivation of using the intervention, which will supplement the qualitative evaluation described below. The knowledge test will focus on key aspects of intrapartum care and will be developed and validated based on a preliminary version from the PartoMa pilot study [23].

##### Qualitative evaluation

As described separately, birth attendants, women giving birth, and hospital leadership will be engaged with different qualitative methods to facilitate a series of task-based investigations [32]. This includes an examination of the barriers and facilitators of the intervention through observations and individual interviews and unpacking collective experiences and perspectives on the strengths and limitations of the PartoMa intervention through FGDs. Together, these investigations will help build a comprehensive understanding of intervention impact and value (key to ‘value for money’) as well as scalability and replicability analyses [56].

##### Evaluation of cost-effectiveness

A pragmatic cost-effectiveness analysis will be conducted from a health-care system and societal perspective, considering incremental costs and effects related to the intervention. Cost data will be collected for both co-creation and implementation. Costs will relate to transport, time, commodities for training, and running and capital costs for training. Data will be collected by participants’ questionnaires and original receipts for direct and indirect costs, as well as daily reports on the activities of facilitators, trainers, and experts throughout co-creation and implementation. Fixed hourly rates based on national salary scales will be used to calculate costs per hour. Capital and running costs per facility and per health worker for both co-creation and implementation will be assessed. All costs will be adjusted by fixed-time exchange rates and purchasing power parity.

Effects will be measured in natural units related to the primary outcome of perinatal deaths (stillbirths with positive foetal heart on admission and pre-discharge neonatal mortality during the first 7 days among inborn babies weighing ≥1000 g) as well as to Apgar score <7. Incremental cost-effectiveness ratios (ICERs) will be calculated separately for stillbirths, neonatal mortality, and Apgar score <7. A discount rate of 3% will be applied to both costs and effect. Changes in stillbirths and neonatal deaths can be transformed into Disability-Adjusted Life Years’ (DALY’s) of a birth cohort by construction of a Markov model to estimate DALYs lost or averted in a lifetime perspective [57]. Finally, ICERs will be calculated using a life-time perspective due to stillbirth and neonatal death for: *i)* natural unit effectiveness measures listed above; and *ii)* modelled DALYs lost or averted [58,59]. Sub-group analysis will be conducted for each of the three facility clusters and for the mode of birth (vaginal versus CS). Base case of costs and effect will be explored in univariate sensitivity analysis, including variation in costs and effects between the five study sites. The non-parametric bootstrap method can be used to estimate sampling distributions of ICER and subsequently compute cost-effectiveness acceptability curves across a range of cost-effectiveness thresholds [57].

#### IV. An enabling CPG development and implementation model

Based on the results of specific objectives I–III and findings from the Zanzibar pilot study, opportunities, and barriers in co-creating, implementing, and upscaling CPGs and training will be analysed. Thereby, the aim is to develop a framework for co-creating and up-scaling context-tailored CPGs and associated implementation strategies [22].

### Power calculation

We applied CCBRT’s routinely collected data for overall births and stillbirths in the five study sites from January to August 2020 (i.e. with the embedded influence of the newly introduced user fee and the COVID-19-pandemic). Based on the newest epidemiological measures from Tanzania (2012), we then set the premise that approximately 50% of stillbirths occurred intrahospital, and, conservatively, that pre-discharge neonatal mortality would be in the same range (i.e. half of overall neonatal mortality) [60]. For each maternity unit, we then calculated the average number of intrahospital perinatal deaths during 3 months, which comprises a block in the stepped-wedged design (Figure 3). Simulation results showed that a power of 80% can be obtained under a relative risk reduction of approximately 22% in intrahospital perinatal deaths, which corresponds to a relative risk (RR) of 0.78. We expect this to be a realistic improvement target when considering concurrent reductions in stillbirths in Zanzibar’s tertiary hospital during the pre-post pilot study of PartoMa, where the majority of the decrease was among intra-hospital stillbirths (overall stillbirth rate fell from 59 to 39 per 1000 total births, RR 0.66; 95% CI 0.53–0.82) [24]. (Supplementary file S1).

Notably, potential declines in facility-births are reflected in these power calculations.

### Data analyses

Overall analyses of the situational findings, co-creation, and implementation processes and effectiveness rely heavily on mixed-methods evaluation. This will include a broad spectrum of designs, from sequential explanatory and exploratory methods to embedded and converging triangulation designs [61].

Concerning the quantitative components, descriptive analyses will be performed in all five facilities as cluster-units of analysis. QoC provision and experience indicators (pre-selected, non-ambiguous criteria) and birth outcomes will be analysed and compared both within and between facilities using mixed effects logistic regression. Differences in seminar attendance and seminar return rates between maternity units will be compared using logistic regression. Knowledge scores are modelled as longitudinal data and compared over time using linear mixed-effects regression. Effects on each outcome will be reported as an average effect across facilities and its significance will be determined by likelihood ratio tests. Possible differences in effect between facilities will be investigated by incorporating a random slope regression model, reported by its standard deviation along with facility-specific deviations from the total effect. Possible time-dependence of the intervention (e.g. delayed entry) is investigated by allowing its effect to depend on facility-specific time since implementation and by adjusting for seasonality patterns. Additional adjustments for covariates will also be performed, such as for staff and patient counts, availability of essential supplies, other competing interventions or unintended events in the facilities, national holidays, maternal characteristics, and referral patterns. Both unadjusted and adjusted results will be reported. A biostatistician in the study team will closely supervise statistical analyses. All summary statistics and effect sizes will be reported along with 95% Wald-based confidence intervals, and statistical significance will be set at 5% level.

The qualitative analysis plan is presented separately [32].

### Ethics

Ethical approval is obtained from the Tanzanian National Institute of Medical Research (NIMR/HQ/R.8a/Vol. IX/3324, NIMR/HQ/R.8c/Vol. I/1679, NIMR/HQ/R.8c/Vol. I/926). Research permits were obtained from the Tanzania Commission of Science and Technology, regional and district medical officers in Dar es Salaam and participating hospitals. A data management agreement has been signed by the partners involved in storing and analysing data. The study is registered in clinicaltrials.gov (NCT04685668).

While we aim to complete the intervention modification process prior to commencing the trial, further modifications may be necessary thereafter, due to emerging evidence, national regulations, or unforeseen errors. Due to the fast-paced stepped-wedged enrolment of the sites (Figure 3), we do not plan for an interim analysis to show the futility of the intervention. Also, as the intervention aims at augmenting established best clinical practices, we do not plan for an interim analysis based on assessments of severe adverse events.

The study team will try its best to avoid taking birth attendants away from their clinical work to participate in co-creation, training, interviews, and other research activities. Steps will be taken to make direct labour observations safe, respectful, and non-blaming, including consideration of observer selection and training in research ethics, development of guidance for observers in case of emergencies, and open and supportive channels of communication and psychological support for observers [62]. Data collectors will not make reminders to staff on minor issues in their clinical care. However, if they suspect a threat to the life of a mother or baby, data collectors are obliged to alert frontline health providers. Further ethical considerations related to the qualitative study components are published separately [32].

Procedures for data storage, data transfer, and handling will follow ethical review board and confidentiality rules in accordance with national regulations in Tanzania and the European Union. The PartoMa Project aims at a participatory quality improvement process to reach the best achievable clinical practice while avoiding exposure and reprimanding of individual birth attendants. All data will be anonymous as codes will replace women’s and health providers’ identities, stored in locked rooms and entered into password-protected preformed electronic databases: quantitative data in the KoBoToolbox software package (https://www.kobotoolbox.org), and qualitative data in OneDrive.

### Dissemination

In addition to publications in peer-reviewed open access journals, dissemination seminars will be held in Tanzania. Milestones and the study's main findings will be shared with facility, regional, national and international stakeholders, including frontline health providers, women who have participated in study parts and the wider community. Central results will also be disseminated at the study website (publichealth.ku.dk/partoma) and shared through the media when possible.

## Key Methodological Strengths and Limitations

This PartoMa Scale-up Study directly addresses the urgent need for research on co-creation, implementation, and scale-up of context-specific CPGs in overstretched, high-volume maternity units in LLMICs [9,22,30]. We have not identified any similar studies.

We developed a pragmatic study design, aiming to produce an extensive description of context, intervention co-creation, and implementation strategy, and include a broad range of process, outcome and economic measures. Such a broad, real-world scale-up may enable an in-depth understanding of how the intervention might achieve impact and may be sustained or translated from one context to another. It, however, also entails inherent trade-offs, of which the most central are presented here [63]:

First, a dilemma arose in deciding the degree to which the intervention can be allowed to vary across the five sites, with limited resources and at the expense of statistical rigor. Unimodal, non-complex interventions do not typically produce lasting changes in complex health-care systems, and we aim to enable flexibility and creativity in the intervention co-creation [63]. However, as we hypothesize co-creation to be highly resource consuming for each single maternity unit in LLMICs, and as health providers are often shifted between the facilities, we plan for one version of the intervention for all five sites, but with some flexibility in implementation plan between sites [22]. In addition, this balance between fidelity and adaptation aims to strengthen the overall trial design, which, if the intervention proves effective, may strengthen the advocacy process for adoption and further scale-up by the government (33). By comparing quantitative and qualitative results between study sites, we may explore potential issues regarding this approach.

Second, low-quality data may influence validity. We have designed a study to overcome this challenge by: *i*. ensuring multiple indicators and research methods at each level of evaluation and thereby the possibility of triangulation; *ii*. exploring routine data monitoring during the situational analysis, which allows measures to be taken to improve the quality of data prior to the trial; *iii*. prospectively collected birth outcome data will be cross-checked regularly; and *iv*. double-entry of data will be applied to the extent resources allow.

Third, it is a key component of the intervention to pragmatically rely on birth attendants’ self-directed learning and motivation (Box 1). While we aim for the modified intervention to closely follow the needs of the attendants, they might not apply it. If pilot testing at the first implementation site leaves us without seminar participants and CPG users, we may have to resume the co-creation process and explore further how to tailor the intervention to local needs and demands. To the contrary, if highly popular among birth attendants, we may experience spill-over where staff from pre-rollout sites attend PartoMa seminars in study sites where the intervention is already implemented. Seminar participants will be registered to assess the extent of this, and contamination will be minimized by the short timeframe between rollout in each facility (Figure 3).

**Table:** 

**Box 1** The PartoMa pilot intervention in Zanzibar, consisting of pocket booklets with locally co-created guidelines on common care during birth and associated quarterly recurring seminars, where the guidelines are practiced in groups passing through five stations. In Zanzibar’s tertiary hospital, a pre-post study suggested that the multifaceted PartoMa intervention appeared associated with a 33% decrease in stillbirths and halving in babies with low Apgar score. The intervention follows a ‘self-directed approach’ where use of the guidelines and participation in seminars are voluntary and based on inner motivation. At each seminar station, locally realistic case stories are discussed, based on partographs and both routine and emergency care during birth, respectful support, triage, and hands-on training are included. The Zanzibar PartoMa guidelines are available online (publichealth.ku.dk/partoma). *Photos by Jurre Rompa, Lara Meguid and Rune Maaløe Andersen. All identifiable people have given their oral consent*.

Finally, a challenge arises if the success of the implementation is highly different across sites, which may leave the overall results of the stepped-wedged trial inconclusive. In this case, the theory-based, mixed-methods study design will allow us to conduct in-depth analyses focused on individual effects at hospital level, which can be compared across sites. This flexibility to assess intra- and inter-site variability is particularly important for addressing questions of transferability and scalability. In addition, while the current evaluation timeframe reflects our funding limitations, it may be argued that an evaluation time frame of nine to 15 months is insufficient for assessing potential long-term impact. If the intervention appears effective and continues, we aim to fundraising for additional post-exit evaluations, including in-depth exploration of long-term sustainability and potential further scale-up in Dar es Salaam and beyond.

## Conclusion

If successful, an enabling CPG development and implementation model for LLMICs that improves health providers’ knowledge, skills, motivation, and clinical care will be of tremendous importance for strengthening maternal and perinatal health, and health systems at large.

## Supplementary Material

Supplemental Material

## Paper context

Research is warranted on co-creation of context-specific clinical guidelines and associated training in low-resource settings. This PartoMa Scale-up Study will assess such a multifaceted intervention’s outputs on multiple levels, with survival during birth as the ultimate outcome. Additionally, it explores barriers and facilitators for co-creation and implementation and assesses cost-effectiveness. If the PartoMa intervention (cost-)effectively improves birth attendants’ abilities to provide safe and respectful care at scale, it may strengthen maternal and perinatal health at large.
