# Supplementary material for: Scaling up context-tailored clinical guidelines and training to improve childbirth care in urban, low-resource maternity units in Tanzania: A protocol for a stepped-wedged cluster randomized trial with embedded qualitative and economic analyses (The PartoMa Scale-Up Study)
Source: Glob Health Action. 2022 Apr 12;15(1):2034135. doi: 10.1080/16549716.2022.2034135 (PMC9009913; doi:10.1080/16549716.2022.2034135)
Supplement: Supplemental Material [file ZGHA_A_2034135_SM0027.pdf]

Power calculation for the PartoMa Study

Andreas Kryger Jensen

24 November, 2021

The modified stepped wedge design of the PartoMa study can be visualized as

```
                     B1  B2  B3  B4  B5  B6
Mbagala Rangi Tatu   0   1   1   1   1   1
Temeke               0   0   1   1   1   1
Sinza                0   0   1   1   1   1
Amana                0   0   0   1   1   1
Mwananymala          0   0   0   1   1   1
```

where each column B1, . . . , B6 denote a block with a duration of three months, and 0/1 denote before and after introducing the intervention respectively. That the order of hospital entries was randomized.

Background data for the power calculation was obtained from the Perinatal Problem Identification (PPI) database maintained by the Comprehensive Community Based Rehabilitation (CCBRT) organization in Tanzania. It consisted of the total number of births and the number of stillbirths at each of the five hospitals (AMN, MNY, MRT, SNZ and TMK) in Dar es Salaam in the period January – August 2020.

Based on the newest epidemiological measures from Tanzania (2012), we then set the premise that approximately 50% of stillbirths occurred intra-hospital, and, conservatively, that pre-discharge neonatal mortality would be in the same range (i.e., half of overall neonatal mortality). For each maternity unit, we then calculated average number of births and intra-hospital perinatal deaths during three months, which comprises a block in the stepped-wedged design. Data for April and May was not available for the AMN hospital and was hence excluded from the average. The averages were extrapolated to the full time period of the study. This will most likely result in a conservative power assessment.

Table 1: Average number of births and intra-hospital perinatal deaths during a three month block from the five hospitals based on the PPI database.

	Average #births/block	Average #intra-hospital perinatal deaths/block	Risk [%]
AMN	1909	61	3.20
MNY	2373	75	3.14
MRT	2866	57	1.99
SNZ	2236	43	1.93
TMK	1594	69	4.33

The power calculation was performed by simulating random data sets under the design and for each simulation calculating the p-value of the treatment effect by a likelihood ratio test in a model adjusting for time (linear effect) and hospital (factor). The power was estimated as the empirical proportion of rejections at the 5% level based on 5,000 independent simulations on a grid of 16 risk ratios between 0.7 and 1.0.

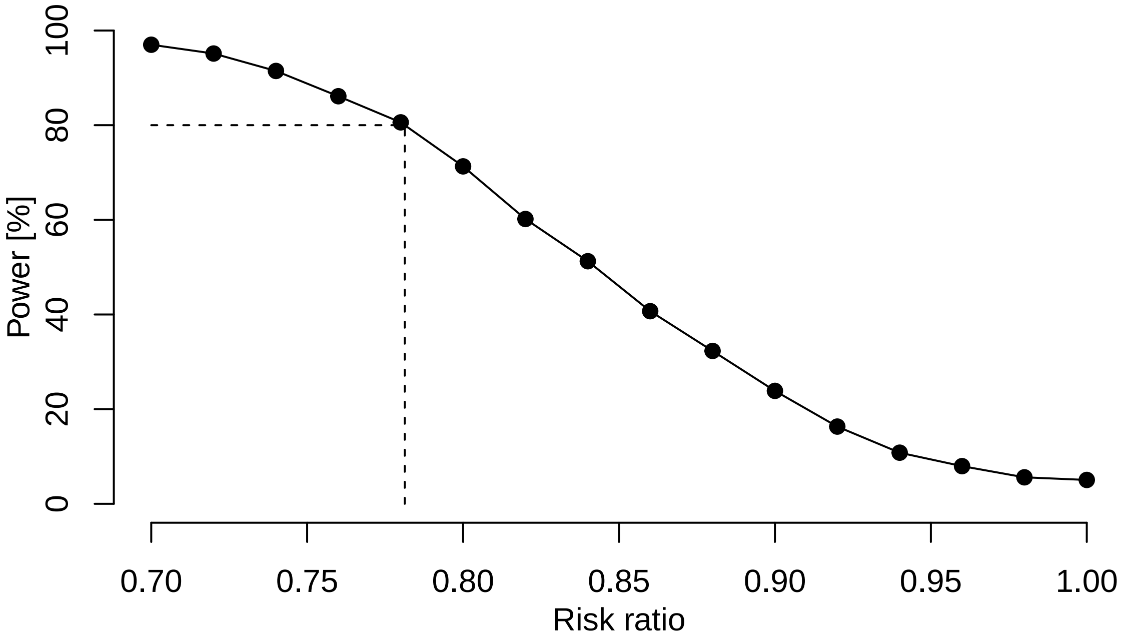

According to the simulations, a power of 80% can be obtained at a relative risk of 0.78 corresponding to a relative reduction in risk of 22%.
